# Naringenin Exhibits Antiglioma Activity Related to Aryl Hydrocarbon Receptor Activity and IL-6, CCL2, and TNF-α Expression

**DOI:** 10.3390/brainsci15030325

**Published:** 2025-03-20

**Authors:** Monique Reis de Santana, Deivison Silva Argolo, Irlã Santos Lima, Cleonice Creusa dos Santos, Maurício Moraes Victor, Gabriel dos Santos Ramos, Ravena Pereira do Nascimento, Henning Ulrich, Silvia Lima Costa

**Affiliations:** 1 Laboratory of Neurochemistry and Cellular Biology, Institute of Health Sciences, Federal University of Bahia, Salvador 40231-300, Brazil; moniquereisant@gmail.com (M.R.d.S.); deivison.argolo@ufba.br (D.S.A.); irlalima@ufba.br (I.S.L.); cleonicemev@gmail.com (C.C.d.S.); ravenanascimento@ufba.br (R.P.d.N.); 2 Department of Organic Chemistry, Institute of Chemistry, Federal University of Bahia, Salvador 40231-300, Brazil; mmvictor@ufba.br (M.M.V.); gabrielramosquimica@gmail.com (G.d.S.R.); 3 Department of Biochemistry, Institute of Chemistry, University of São Paulo, São Paulo 05508-220, Brazil; henning@iq.usp.br; 4 National Institute of Translational Neuroscience (INNT), Rio de Janeiro 21941-902, Brazil

**Keywords:** glioblastoma, AhR, flavonoid

## Abstract

Background: Glioblastoma (GBM) is a highly aggressive brain tumor characterized by rapid cell proliferation, invasive behavior, and chemoresistance. The aryl hydrocarbon receptor (AhR) is implicated in chemoresistance and immune evasion, making it a promising therapeutic target. Natural compounds such as flavonoids have gained attention for their anti-inflammatory, antioxidant, and anticancer properties. Among them, naringenin, a citrus-derived flavonoid, exerts antiproliferative, pro-apoptotic, and immunomodulatory effects. Objectives: This study investigated the antiglioma effects of the flavonoid naringenin on the viability, growth, and migration of glioma cells and its potential role as an AhR modulator. Methods: Human (U87) and rat (C6) glioma cell lines were exposed to naringenin (10–300 µM) alone or in combination with the AhR agonist indole-3-carbinol (50 µM) for 24 to 48 h. Cell viability, scratch wound, and cell migration assays were performed. The expression of inflammatory markers was also analyzed by RT-qPCR. Results: Naringenin exerted dose- and time-dependent inhibition of cell viability and migration. The treatment decreased the gene expression of interleukin-6 (IL-6) and chemokine (CCL2), alongside increased tumor necrosis factor-alpha (TNF-α) expression, an effect reversed by the AhR agonist. Conclusions: These findings highlight naringenin’s potential as an antiglioma agent and its role in AhR signaling.

## 1. Introduction

Glioblastoma (GBM) is one of the most aggressive and malignant primary brain tumors [1,2]. It is characterized by high cellular proliferation and infiltrative, invasive properties, resulting in a high recurrence rate despite standard treatment, which involves maximal safe resection followed by radiation therapy and temozolomide (TMZ) chemotherapy [3,4,5]. The median survival for GBM patients is approximately 14 months, with treatment protocols remaining largely unchanged over the past two decades [6]. Given these challenges, there is a pressing need to identify novel therapeutic targets and develop more effective treatment strategies for GBM [7].

Many mechanisms contribute to tumor progression and immunosuppression in GBM, including metabolic remodeling in glioma and immune cells [8]. The aryl hydrocarbon receptor (AhR) is a transcription factor that regulates genes involved in diverse physiological processes, such as development, immune responses, and metabolism [9]. Its activity has been strongly correlated with poor cancer outcomes. The AhR plays a pivotal role in driving metabolic reprogramming in tumor cells by modulating genes associated with proliferation, migration, invasion, immunosuppression, and resistance to chemotherapy and immunotherapy [10,11,12]. Growing evidence suggests that AhR activation promotes tumor cell migration and contributes to drug resistance in breast and pancreatic cancer and GBM [11,13,14]. The receptor is overexpressed and constitutively active in glioblastoma. A study by Opitz, in 2011 [15], detected that the constitutive activation of the AhR, driven by increased kynurenine (Kyn) production, has been shown to contribute to glioma malignancy and progression. There is strong evidence that dysregulated tryptophan metabolism in GBM promotes glioma progression via AhR activation and downstream signaling [16]. Studies demonstrated that AhR activation, induced by tryptophan metabolism and Kyn accumulation, enhances tumor cell proliferation and invasion in patient-derived glioma samples [17] and human GBM cell lines [18], findings that highlight the AhR as a promising therapeutic target for glioblastoma treatment.

Flavonoids have been extensively studied for their anticancer potential [19]. These polyphenolic compounds, abundant in fruits and vegetables, exert antitumor effects through antioxidant activity and the modulation of key signaling pathways involved in tumor progression [20]. Naringenin, a flavanone primarily found in citrus fruits [21], has demonstrated regulatory effects on molecular pathways associated with various cancers, including lung [22], breast [23,24], and skin [25] cancers and GBM [26]. Stompor et al. (2017) [27] investigated the antiglioma properties of naringenin after a 24 h treatment using a glioma cancer cell line (U118MG). In this study, naringenin demonstrated six-fold higher selectivity for GBM cells than normal fibroblasts. Naringenin has also been shown to inhibit glioblastoma cell migration and invasion by downregulating MMPs (2 and 9) and suppressing the activity of ERK1/2 and p38 [28]. More recently, it was suggested that naringenin modulates proliferation, metastasis, and glioma cell migration by regulating the Hedgehog (Hh) signaling pathway [29]. Recently, we characterized the antagonism of the AhR by naringenin in cancer cells and also the amino acid residues of the binding domain in silico [30]. A recent study by Yan et al. (2023) [31] showed that naringenin inhibits the activation of the NLRP3 inflammasome, promoting AhR nuclear translocation and increasing its expression in vivo. On the other hand, studies have demonstrated that IL-6 and TNF-α contribute to glioma cell proliferation, invasion, and migration [32,33]. Additionally, CCL2 overexpression enhances cancer cell migration and recruits immunosuppressive cells to the tumor microenvironment, creating conditions that support tumor progression, and its elevated expression is associated with a poor prognosis in GBM patients [34]. Despite these findings, the antiglioma effects and regulatory mechanisms of naringenin via the AhR and the modulation of inflammatory factors related to immunoresistance remain largely unexplored, highlighting the need for further investigation to better understand its potential therapeutic role.

This study evaluated naringenin’s impact on glioma cells, with a focus on its potential to modulate AhR activity and the regulation of key inflammatory mediators involved in tumor proliferation, migration, and chemoresistance.

## 2. Materials and Methods

### 2.1. Tumor Cell Cultures

U87 human GBM and C6 rat glioma cells were cultured in Dulbecco’s Modified Eagle Medium (DMEM; Island Biological Company-GIBCO^®^, Grand Island, NY, USA), containing 7 mmol/L glucose, 2 mmol/L L-glutamine, and 0.011 g/L pyruvic acid supplemented with 10% fetal bovine serum (FBS) and antibiotics (100 U/mL penicillin and 100 μg/mL streptomycin; Gibco^®^, Grand Island, NY, USA), and maintained in an incubator under standardized conditions in a humidified atmosphere with 5% CO_2_ at 37 °C, as described previously [35]. Cells were cultured in 100 mm polystyrene plates (TPP, Trasadingen, Switzerland). Upon reaching confluence, the medium was removed. Adherent cells were detached by using a trypsin solution (0.05% trypsin and 0.02% EDTA in PBS) and seeded into 6- or 96-well polystyrene plates (Kasvi, São José dos Pinhais, Brazil), according to the experiment, at a density of 8 × 104 cells/cm^2^.

### 2.2. Spheroid Formation

To prepare agarose casts, a 2% agarose solution (1 g of agarose in 50 mL of 0.9% NaCl solution) was autoclaved along with rubber molds. After autoclaving, the molten agarose was cooled, under aseptic conditions in a cell culture hood, and 500 µL of agarose was pipetted into an 81-microwell rubber mold. Once the agarose had solidified, the rubber molds were flexed carefully to release the agarose casts directly into the wells of a 12-well plate. Agarose casts were equilibrated by adding cell culture medium (2.5 mL/well) and incubated at 37 °C with 5% CO_2_ for one hour. For cell seeding, the appropriate number of cells per agarose cast was prepared, with 275,000 cells for an 81-microwell cast, with an average of 1000 cells per spheroid. The culture medium surrounding the agarose casts was removed with a P1000 pipette, and the cell suspension was seeded dropwise into the agarose microwells. After seeding, the well plate was returned to the incubator for 15 min to allow the cells to settle into the microwells. Additional culture medium (2.5 mL/well for 12-well plates) was added, and the plates were incubated for 48 h. Spheroids were allowed to form and stabilize over this period, and if necessary, the culture medium was carefully replaced by tilting the plate and using a pipette to remove the surrounding medium.

### 2.3. Drugs and Treatments

The flavonoid naringenin (NAR) was obtained from the hydrolysis of naringin extracted from the peel of *Citrus paradisi* (Grapefruit) [36] and from (S)-naringenin obtained based on tests carried out by Apigenin. 4′, 5,7-Trihydroxy flavone was prepared from the extracted naringin [37]. The compound indole-3-carbinol (I3C), an AhR agonist, was purchased from Sigma (I3C; I7256—Sigma, Saint Louis, MO, USA). The structure of these molecules is shown in Table 1. The molecules were dissolved in dimethyl sulfoxide (DMSO; Sigma, Tokyo, Japan) to form a 100 mM stock solution, which was stored and protected from light at −4 °C. When applied to cells, flavonoid naringenin and agonists were diluted directly in a culture medium (DMEM) without fetal bovine serum (FBS). Treatment of cells with the vehicle DMSO was adopted as control, which did not show a significant effect on the analyzed parameters when compared with cultures that were not exposed to this solvent.

### 2.4. Cell Viability Assays

Metabolic activity as a measure of the viability of U87 and C6 cell lines was assessed by using the 3-(4,5-dimethylthiazol-2-yl)-2,5-diphenyltetrazolium bromide (MTT) test and the sulforhodamine B (SRB) assay for cell density measurements as described previously [38,39,40].

#### 2.4.1. MTT Assay

For the MTT assay, cell viability was determined by the conversion of the yellow salt (MTT) into formazan crystals (purple) by the dehydrogenases of live cells. Glioma cells (U87 and C6) were seeded in 96-well plates (Kasvi^®^, Pinhais, Brazil) at a density of 2.2 × 10^4^ cells/cm^2^ (8000 cells/well) in DMEM supplemented with FBS at 37 °C for 24 h. The cells were treated under the control condition or with previously defined concentrations of naringenin diluted in DMEM without SFB and incubated for 24 h. After treatments, cells were incubated with an MTT solution (Thermo Fisher, Waltham, MA, USA; 0.5 mg of MTT per 1 mL) at 37 °C and 5% (*v/v*) CO_2_ for 2 h. Next, 100 μL of a lysis buffer containing 20% (*w/v*) sodium dodecyl sulfate (SDS), 50% (*v/v*) acetic acid, and 2.5% (*v/v*) 1 mol/L HCl was added. The plates were incubated overnight, and absorbance at 570 nm was determined by using a microplate reader (Varioskan™ LUX multimode microplate reader). Three separate experiments were performed, each with eight replicates per variable. The results were presented as the percentage of viability of the treated groups compared with the control, which was considered 100%.

#### 2.4.2. Sulforhodamine B (SRB) Assay

Cell viability by the SRB assay was determined by the uptake and binding of a bright pink dye by basic amino acids, which dissociate under basic conditions, under acidic conditions. After treatment, cells were fixed by adding 100 μL of 10% trichloroacetic acid (TCA) per well and stored at 4 °C for a minimum of 1 h. TCA was rinsed four times with deionized water (100 μL/well), and 100 μL of SRB (S1307, Invitrogen, Waltham, MA, USA) solution was added into each well of the culture plate for 30 min. After incubation, cells were rinsed four times with 100 μL of 1% glacial acetic acid to remove the unbound dye, and the plates were allowed to dry at room temperature. The solubilization of the protein-bound dye was realized through the pipetting of 200 μL of 10 mM Tris-base at pH 10.5 (T6791, Sigma) per well. Absorbance was measured at 690 nm by using the microplate reader (Varioskan™ LUX multimode microplate reader, Thermofisher Scientifics, Waltham, MA, USA). Three independent experiments were performed, and the relative viability rate was determined based on the absorbance under each condition, considering control to be 100%.

### 2.5. Migration Assays

#### 2.5.1. Monolayer Scratch Assay

The potential of the flavonoid naringenin for inhibiting cell migration was investigated in a single monolayer lesion test or scratch assay [41]. U87 cells were seeded into 6-well plates (Kasvi^®^, Weissópolis, Pinhais, Brazil) at a density of 5 × 10^5^ cells per well and cultured for 24 h to form a confluent monolayer. A scratch was introduced through the cell monolayer by using a 200 μL pipette tip, forming a gap as a straight line in one direction. The cell debris was removed by washing the wells twice with phosphate-buffered saline (PBS). The cultures were then incubated with serum-free medium containing either naringenin (30 µM), indole-3-carbinol (I3C; 50 µM), a combination of both, or a control condition (0.1% DMSO). Migration into the scratched area was assessed after 24 and 48 h, and images of each sample were captured from the same location, at the center of the well, under identical lighting and magnification conditions. Images were captured by an optical phase microscope (Nikon TS-100, Nikon, Melville, NY, USA) equipped with a digital camera (Nikon E4300, Nikon). The migration rate was assessed by the number of cells that migrated into the area of the scratch, which was precisely defined at the 0 h time point. Migration was quantified by counting the cells inside defined gaps over time by using ImageJ software v 1.53t (2024) (Wayne Rasband, National Institutes of Health, Bethesda, MD, USA). All experiments were performed in triplicate.

#### 2.5.2. Spheroid-Based Migration Assay

U87 glioma spheroids were prepared by seeding cells into 96-well plates designed for spheroid formation and incubated for 7 days to allow for spheroid stabilization. After this period, the spheroids were transferred to a flat, adherent 96-well plate and exposed to either naringenin (30 µM) or maintained under the control condition (0.1% DMSO). The migratory capacity of the cells from the spheroids was assessed after 24 and 48 h by capturing phase-contrast images. The quantification of migrating cells from the spheroid was performed. The migration rate was quantified by using ImageJ software (Wayne Rasband, National Institutes of Health, Bethesda, MA, USA). All experiments were performed in triplicate.

### 2.6. RT-qPCR for mRNA

C6 cells were cultured in 6-well plates (Kasvi, Pinhais, Brazil) at a cell density of approximately 1 × 10^5^ cells/cm^2^ and maintained under control conditions (0.03% DMSO) or exposed to naringenin (30 µM) and/or I3C (30 µM) for 24 h. After 24 h of treatments, total RNA was extracted by using Trizol^®^ reagent (Thermo Fisher Scientific, Waltham, MA, USA) following the manufacturer’s recommended protocol. RNA quantification was performed by using NanoDrop Lite (Thermo Fisher Scientific). For the cDNA reaction, 5 μg of RNA was used with the GoScript high-capacity cDNA reverse transcription kit, following the manufacturer’s instructions (Promega, Madison, WI, USA). The cDNA was stored at −20 °C until use. Subsequently, quantitative real-time PCR (RT-qPCR) was performed by using the QuantStudio 12k instrument (Applied Biosystems, Waltham, MA, USA) under standard TaqMan thermal cycling conditions as per the manufacturer’s instructions. mRNA expression was evaluated by using commercial TaqMan^®^ probes: IL-6 (Rn01410330_m1), TNF (Rn99999017_m1), and CCL2 (Rn00580555_m1). The reference gene, HPRT (Rn01527840_m1), was chosen as a normalizer due to its validation as a reliable reference gene for mRNA expression in contexts where AhR pathway transcription is altered in rats and mice [42,43]. mRNA expression levels were calculated by using the 2^−∆∆CT^ method [44] and analyzed by using GraphPad Prism v 9.1.1 (2020) (La Jolla, CA, USA) for Mac. The results represent at least three independent experiments performed.

### 2.7. Statistical Analysis

Data were statistically analyzed by using GraphPad Prism v 9.1.1 2020 (La Jolla, CA, USA). Results in each experimental group are presented as means ± standard errors (SEMs). The results were analyzed by one-way analysis of variance (ANOVA), and experimental groups were compared in pairs by using the Mann–Whitney post-test. Values of *p* < 0.05 were considered statistically significant. All experiments were repeated at least three times.

## 3. Results

### 3.1. Naringenin Alters the Viability of Glioma Cell Lines U87 and C6

**Figure 1:**
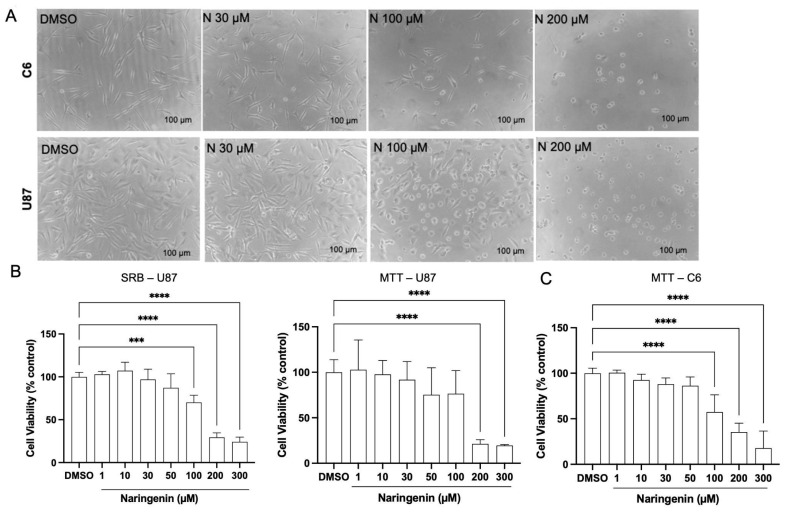
Effects of naringenin (N) on viability of rat (C6) and human (U87) GBM cells. (**A**) MTT and SRB assay analyses of U87 cell viability and density after 24 h. (**B**) MTT assay analysis of C6 cell viability after 24 h. (**C**) Phase-contrast photomicrographs of C6 and U87 cell cultures exposed to naringenin (N; 30–200 µM) and the control condition with DMSO (0.3%) after 24 h; scale bar = 100 μm. Results are expressed as the average percentage of viable cells, considering the control condition to be 100% ± SEM (*n* = 3). The asterisks (*) on the bars indicate a statistical difference relative to the control, as determined by the Mann–Whitney post-test: **** *p* <0.0001; *** *p* <0.001.

**Figure 2:**
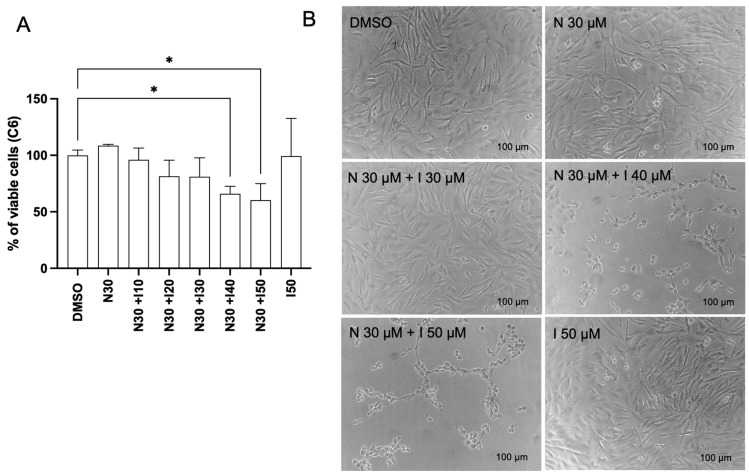
Effects of naringenin (N) and indole-3-carbinol (I) on viability of rat (C6) GBM cells. (**A**) SRB assay analysis of C6 cell viability after 24 h. (**B**) Phase-contrast photomicrographs of C6 cell cultures exposed to N (30 µM), I (10–50 µM), and the control condition with DMSO (0.05%) after 24 h; scale bar = 100 μm. Results are expressed as the average percentage of viable cells, considering the control condition to be 100% ± SEM (*n* = 3). The asterisk (*) on the bars indicates a statistical difference relative to the control, as determined by the Mann–Whitney post-test: * *p* < 0.05.

The cytotoxic effects of naringenin on rat (C6) and human (U87) glioma cells were assessed by using MTT and SRB assays. The 50% inhibitory concentration (IC50) was determined from the viability assays, revealing IC50 values exceeding 100 μM. Both assays demonstrated comparable cytotoxic effects of naringenin on U87 cells. As shown in Figure 1A, naringenin significantly reduced cell viability (*p* < 0.05) 24 h after exposure compared with the control group (3% DMSO), with IC50 values of 129.4 µM (MTT) and 132.3 µM (SRB). In C6 cells, naringenin also caused a significant, dose-dependent reduction in viability (IC50 = 150.9 µM) and induced noticeable morphological changes, including decreased cell adhesion (Figure 1B,C). Furthermore, the viability of C6 cells exposed to a subtoxic concentration of naringenin (30 µM) in combination with the AhR agonist indole-3-carbinol (I3C) (10–50 µM) was investigated by the SRB assay. The results showed a significant cytotoxic effect of co-treatments at concentrations of I3C above 40 µM, while I3C alone (50 µM) did not affect cell viability (Figure 2).

### 3.2. Naringenin Inhibits the Migration of Glioma Cells

**Figure 3:**
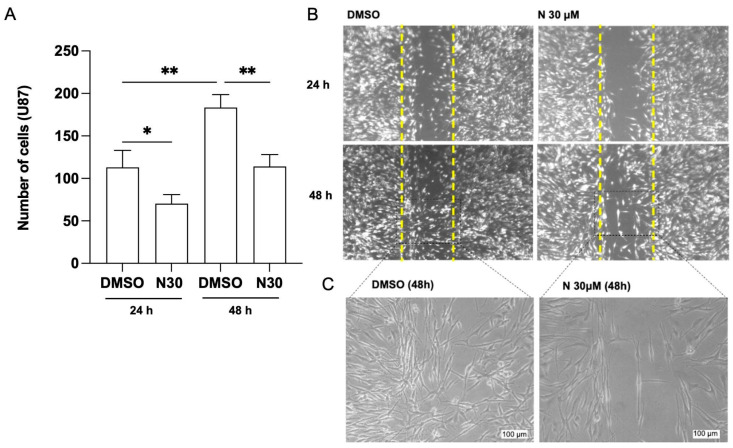
Naringenin (NAR) reduced migration of glioblastoma cells (U87). (**A**) Quantification of number of cells migrated into scratch area, 24 and 48 h after exposure. (**B**) Phase-contrast photomicrographs of U87 cell cultures exposed to 30 µM NAR and control condition (0.05% DMSO), representing the scratch area 24 and 48 h after exposure; scale bar = 100 μm. (**C**) Enlarged phase-contrast photomicrographs of the edges, 48 h after exposure to 30 µM NAR and control condition (0.05% DMSO); scale bar = 100 μm. Yellow dashed lines indicate the initial scratch area. Migratory cells were quantified by using ImageJ 24 and 48 h after exposure. Results are expressed as the average number of viable cells within the lesion area 24 and 48 h after exposure and compared with the control. The asterisks (*) on the bars indicate a statistical difference relative to the control, as determined by the Mann–Whitney post-test: * *p* < 0.05; ** *p* < 0.01.

For assessing the anti-invasive potential of naringenin in GBM cells, the inhibition of U87 cell migration was evaluated in monolayer migration assays. The results revealed that naringenin at 30 µM caused a significant delay in U87 cell migration toward the monolayer gap, particularly 48 h after exposure, compared with the control conditions (0.05% DMSO) (Figure 3A,B).

**Figure 4:**
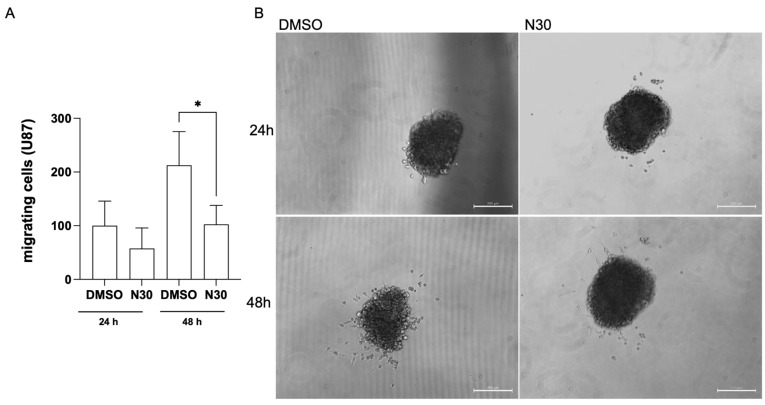
Naringenin (N30) decreased flat surface cell migration from U87 spheroids. (**A**) Quantification of viable cells migrated from spheroids to plate flat surface, 24 and 48 h after exposure. (**B**). Representative phase-contrast photomicrographs of U87 spheroids exposed to naringenin at a concentration of 30 µM and control condition (0.1% DMSO); scale bar = 200 μm. Migrating cells were quantified by using ImageJ. Data are expressed as mean values  ±  SEMs (n = 3). Results are expressed as the average number of viable cells migrated from the spheroid. The asterisk (*) on the bars indicates a statistical difference relative to the control, as determined by the Mann–Whitney post-test: * *p* < 0.05.

Naringenin (30 µM) significantly inhibited the migration of single cells from U87 spheroids to a flat surface (adherent plate). Cell migration from the spheroids 24 and 48 h after naringenin exposure compared to the control (0.3% DMSO) was markedly reduced in number (Figure 4).

### 3.3. Antagonistic Potential of Naringenin in Regulating AhR Target Gene Expression in Glioma Cells

**Figure 5:**
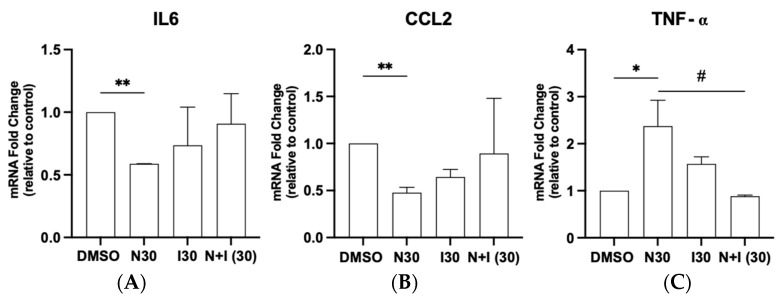
Effect of naringenin in association or not with AhR agonist on mRNA expression of IL-6 (**A**), CCL2 (**B**), and TNF-α (**C**) in rat glioma cells (C6). C6 cells were treated with naringenin at a concentration of 30 µM (N30) alone or in combination with indole-3-carbinol at a concentration of 30 µM (I30) or maintained under control conditions (0.03% DMSO). Cytokine expression was analyzed by RT-qPCR after 24 h treatments. Data are expressed as means ± SEMs (n = 3) of mRNA expression relative to the control. The asterisks (*) on the bars indicate a statistical difference relative to the control, and the hash (#) indicates statistical differences between groups, as determined by the Mann–Whitney post-test: * *p* < 0.05; ** *p*< 0.01; # *p* < 0.05.

We hypothesized that naringenin would affect the expression levels of inflammatory markers. Under subtoxic conditions (30 µM), naringenin was efficient in reducing mRNA expression of IL-6 and CCL2 compared with the control (Figure 5A,B) but increased TNF-α expression levels (Figure 5C). To further investigate the role of naringenin in regulating IL-6, CCL2, and TNF-α expression, with particular emphasis on its ability to modulate the AhR, glioma cells were co-treated with the AhR agonist I3C (30 µM). Co-exposure to naringenin and I3C (30 µM) restored IL-6, TNF-α, and CCL2 expression to levels similar to the control, highlighting naringenin’s capacity to influence these cytokines through AhR modulation.

## 4. Discussion

Naringenin, a flavonoid found in citrus fruits, demonstrates a variety of biological and pharmacological activities, with a particular emphasis on its anticancer effects [45]. Although the antiglioma properties of naringenin are recognized [26,29], the detailed molecular mechanisms remain unknown. In this study, naringenin effectively reduced the viability of human glioma cells (U87) and rat glioma cells (C6). The findings of this study align with previous research that showed a notable concentration-dependent decrease in GBM 8401 and C6 cell viability at similar concentrations adopted in this study [29,46].

The AhR signaling pathway has been implicated in a variety of cellular processes that play a significant role in tumor pathogenesis, including the regulation of cell proliferation, cell cycle control, the modulation of adhesion, and the regulation of cell migration [47,48]. Studies highlight the role of the AhR in regulating cell mobility and demonstrate that inhibiting the AhR in glioma blocks the invasive activity of these cells [11,49]. Among the characteristics of malignant gliomas that are associated with the challenges to effective treatment, the high migratory activity of these cells stands out, contributing to rapid dissemination and infiltrative growth [50,51]. Thus, targeting and reducing migratory behavior may enhance therapeutic outcomes. As observed in previous studies, naringenin has the potential to inhibit the migratory activity of C6 cells [26,29]. Furthermore, we have previously demonstrated that the non-toxic concentration of naringenin (30 µM) effectively modulates AhR activity in vitro [30]. Hence, we investigated whether naringenin could affect the migration activity of human glioma U87 cells. We observed that naringenin significantly reduced the migration ability of cells as evidenced in both 2D and 3D models, highlighting its potential as a modulator of tumor cell movement.

We also investigated the effects of naringenin on modulating the inflammatory molecules IL-6, CCL2, and TNF-α associated with immunosuppression in glioma cells. We observed that naringenin at 30 µM significantly reduced IL-6 mRNA expression compared with the control. Notably, previous studies have demonstrated that inhibiting IL-6 signaling directly affects glioma cell proliferation in U87 cells and GBM explant cultures [52]. Given the critical role of inflammatory cytokines in the glioblastoma tumor microenvironment (TME), we examined CCL2, another key mediator of tumor progression. Elevated CCL2 levels correlate with a poor prognosis in GBM patients [53]. Notably, our findings reveal that naringenin treatment reduced CCL2 mRNA transcription, suggesting its potential to restrain glioma development and progression. Consistently, studies have shown that suppressing CCL2 expression in vitro and in vivo effectively inhibited GBM cell growth [54]. On the other hand, we observed an increase in mRNA transcript for TNF-α. This chemokine plays a key role in regulating inflammation within the TME and facilitating the recruitment of peripheral immune cells by GBM cells, potentially contributing to tumor progression [55]. Zhu et al. (2022) [33] demonstrated that low concentrations of TNF-α stimulated cell proliferation, whereas higher concentrations of TNF-α slowed down cell proliferation and potentially induced apoptosis. Moreover, exploring the mechanisms underlying naringenin’s antiglioma effects, we co-treated N30 and the AhR agonist indole-3-carbinol at non-toxic concentrations, and the mRNA expression of IL-6, CCL2, and TNF-α was also analyzed. We observed that IL-6, CCL2, and TNF-α expression levels were restored to those observed under control conditions, reinforcing that naringenin may exert its modulatory effects through interaction with the AhR, thereby influencing the expression of these genes. These findings highlight the complexity of naringenin’s antitumor effects and underscore the need to consider multiple regulatory mechanisms in future studies. While we did not assess AhR expression modulation in this study, future research should investigate how additional factors may impact AhR expression and signaling pathways in glioma cells.

## 5. Conclusions

The results of the present study, along with previous research, confirm the antiglioma potential of the flavonoid naringenin, showing its ability to inhibit migratory and invasive activities in glioma cell cultures and spheroids. Furthermore, it supports the hypothesis that naringenin may have a therapeutic role by interacting with the AhR and modulating the expression levels of its target genes. Although these findings support the role of naringenin in modulating AhR activity, further comprehensive investigations are required to fully elucidate its mechanisms. These findings underscore the complexity of naringenin’s molecular mechanisms and highlight AhR signaling as a potential underlying mechanism for naringenin’s anticancer effects in malignant glioma cells.

## Figures and Tables

**Table 1:** Structural models of tested molecules. Models generated by Molview v2.4.

Molecule	Structure
Naringenin	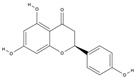
Indole-3-carbinol	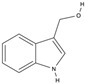

## Data Availability

The original data presented in the study are included in the article.

## References

[1] Miller K.D., Ostrom Q.T., Kruchko C., Patil N., Tihan T., Cioffi G., Fuchs H.E., Waite K.A., Jemal A., Siegel R.L. (2021). Brain and other central nervous system tumor statistics. CA A Cancer J. Clin..

[2] Price M., Ballard C., Benedetti J., Neff C., Cioffi G., Waite K.A., Kruchko C., Barnholtz-Sloan J.S., Ostrom Q.T. (2024). CBTRUS Statistical Report: Primary Brain and Other Central Nervous System Tumors Diagnosed in the United States in 2017–2021. Neuro Oncol..

[3] Du L., Xing Z., Tao B., Li T., Yang D., Li W., Zheng Y., Kuang C., Yang Q. (2020). Both IDO1 and TDO contribute to the malignancy of gliomas via the Kyn–AhR–AQP4 signaling pathway. Signal Transduct. Target. Ther..

[4] Obrador E., Moreno-Murciano P., Oriol-Caballo M., López-Blanch R., Pineda B., Gutiérrez-Arroyo J.L., Loras A., Gonzalez-Bonet L.G., Martinez-Cadenas C., Estrela J.M. (2024). Glioblastoma Therapy: Past, Present and Future. Int. J. Mol. Sci..

[5] Louis D.N., Perry A., Wesseling P., Brat D.J., Cree I.A., Figarella-Branger D., Hawkins C., Ng H.K., Pfister S.M., Reifenberger G. (2021). The 2021 WHO classification of tumors of the central nervous system: A summary. Neuro-Oncology.

[6] Chang C., Chavarro V.S., Gerstl J.V.E., Blitz S.E., Spanehl L., Dubinski D., Valdes P.A., Tran L.N., Gupta S., Esposito L. (2024). Recurrent Glioblastoma—Molecular Underpinnings and Evolving Treatment Paradigms. Int. J. Mol. Sci..

[7] Cruz J.V.R., Batista C., Afonso B.d.H., Alexandre-Moreira M.S., Dubois L.G., Pontes B., Neto V.M., de Mendes F.A. (2022). Obstacles to Glioblastoma Treatment Two Decades after Temozolomide. Cancers.

[8] Erices J.I., Bizama C., Niechi I., Uribe D., Rosales A., Fabres K., Navarro-Martínez G., Torres Á., San Martín R., Roa J.C. (2023). Glioblastoma Microenvironment and Invasiveness: New Insights and Therapeutic Targets. Int. J. Mol. Sci..

[9] Mescher M., Haarmann-Stemmann T. (2018). Modulation of CYP1A1 metabolism: From adverse health effects to chemoprevention and therapeutic options. Pharmacol. Ther..

[10] Takenaka M.C., Gabriely G., Rothhammer V., Mascanfroni I.D., Wheeler M.A., Chao C.C., Gutiérrez-Vázquez C., Kenison J., Tjon E.C., Barroso A. (2019). Control of tumor-associated macrophages and T cells in glioblastoma via AHR and CD39. Nat. Neurosci..

[11] Liu Y., Chen Y., Sha R., Li Y., Xu T., Hu X., Xu L., Xie Q., Zhao B. (2021). A new insight into the role of aryl hydrocarbon receptor (AhR) in the migration of glioblastoma by AhR-IL24 axis regulation. Environ. Int..

[12] Paris A., Tardif N., Galibert M.D., Corre S. (2021). AhR and cancer: From gene profiling to targeted therapy. Int. J. Mol. Sci..

[13] Soltani-asl M., Azimnasab-sorkhabi P., Yoshinaga T.T., de Oliveira Massoco C., Kfoury J.R. (2024). The combination of IDO and AHR blockers reduces the migration and clonogenicity of breast cancer cells. Immunol. Res..

[14] Jin U.H., Kim S.B., Safe S. (2015). Omeprazole Inhibits Pancreatic Cancer Cell Invasion through a Nongenomic Aryl Hydrocarbon Receptor Pathway. Chem. Res. Toxicol..

[15] Opitz C.A., Litzenburger U.M., Sahm F., Ott M., Tritschler I., Trump S., Schumacher T., Jestaedt L., Schrenk D., Weller M. (2011). An endogenous tumour-promoting ligand of the human aryl hydrocarbon receptor. Nature.

[16] Obara-Michlewska M. (2022). The tryptophan metabolism, kynurenine pathway and oxidative stress—Implications for glioma pathobiology. Neurochem. Int..

[17] Zhong C., Peng L., Tao B., Yin S., Lyu L., Ding H., Yang X., Peng T., He H., Zhou P. (2022). TDO2 and tryptophan metabolites promote kynurenine/AhR signals to facilitate glioma progression and immunosuppression. Am. J. Cancer Res..

[18] Ma W., Ye L., Zhong C., Li J., Ye F., Lv L., Yu Y., Jiang S., Zhou P. (2022). Kynurenine produced by tryptophan 2,3-dioxygenase metabolism promotes glioma progression through an aryl hydrocarbon receptor-dependent signaling pathway. Cell Biol. Int..

[19] Pereira do Nascimento R., Lino dos Santos B., Alves Oliveira Amparo J., Ribeiro Pereira Soares J., Costa da Silva K., Reis Santana M., Maria Alves Nunes Almeida Á., Diógenes Amaral da Silva V., de Fátima Dias Costa M., Ulrich H. (2021). Neuroimmunomodulatory Properties of Flavonoids and Derivates: A Potential Action as Adjuvants for the Treatment of Glioblastoma. Pharmaceutics.

[20] Wong S.C., Kamarudin M.N.A., Naidu R. (2023). Anticancer Mechanism of Flavonoids on High-Grade Adult-Type Diffuse Gliomas. Nutrients.

[21] Roszkowski S. (2023). Application of Polyphenols and Flavonoids in Oncological Therapy. Molecules.

[22] Lu W.L., Yu C.T.R., Lien H.M., Sheu G.T., Cherng S.H. (2020). Cytotoxicity of naringenin induces Bax-mediated mitochondrial apoptosis in human lung adenocarcinoma A549 cells. Environ. Toxicol..

[23] Zhao Z., Jin G., Ge Y., Guo Z. (2019). Naringenin inhibits migration of breast cancer cells via inflammatory and apoptosis cell signaling pathways. Inflammopharmacology.

[24] Qi Z., Kong S., Zhao S., Tang Q. (2021). Naringenin inhibits human breast cancer cells (MDA-MB-231) by inducing programmed cell death, caspase stimulation, G2/M phase cell cycle arrest and suppresses cancer metastasis. Cell. Mol. Biol..

[25] Choi J., Lee D.H., Jang H., Park S.Y., Seol J.W. (2020). Naringenin exerts anticancer effects by inducing tumor cell death and inhibiting angiogenesis in malignant melanoma. Int. J. Med. Sci..

[26] Jayalakshmi J., Vanisree A.J. (2020). Naringenin Sensitizes Resistant C6 Glioma Cells with a Repressive Impact on the Migrating Ability. Ann. Neurosci..

[27] Stompor M., Uram Ł., Podgórski R. (2017). In vitro effect of 8-prenylnaringenin and naringenin on fibroblasts and glioblastoma cells-cellular accumulation and cytotoxicity. Molecules.

[28] Chen Y.Y., Chang Y.M., Wang K.Y., Chen P.N., Hseu Y.C., Chen K.M., Yeh K.T., Chen C.J., Hsu L.S. (2019). Naringenin inhibited migration and invasion of glioblastoma cells through multiple mechanisms. Environ. Toxicol..

[29] Sargazi M.L., Juybari K.B., Tarzi M.E., Amirkhosravi A., Nematollahi M.H., Mirzamohammdi S., Mehrbani M., Mehrabani M., Mehrabani M. (2021). Naringenin attenuates cell viability and migration of C6 glioblastoma cell line: A possible role of hedgehog signaling pathway. Mol. Biol. Rep..

[30] de Santana M.R., dos Santos Y.B., Santos K.S., Junior M.C.S., Victor M.M., Ramos G.d.S., Nascimento R.P.D., Costa S.L. (2024). Differential Interactions of Flavonoids with the Aryl Hydrocarbon Receptor In Silico and Their Impact on Receptor Activity In Vitro. Pharmaceuticals.

[31] Yan X., Lin T., Zhu Q., Zhang Y., Song Z., Pan X. (2023). Naringenin protects against acute pancreatitis-associated intestinal injury by inhibiting NLRP3 inflammasome activation via AhR signaling. Front. Pharmacol..

[32] West A.J., Tsui V., Stylli S.S., Nguyen H.P.T., Morokoff A.P., Kaye A.H., Luwor R.B. (2018). The role of interleukin-6-STAT3 signalling in glioblastoma. Oncol. Lett..

[33] Zhu X., Shi G., Lu J., Qian X., Wang D. (2022). Potential regulatory mechanism of TNF-α/TNFR1/ANXA1 in glioma cells and its role in glioma cell proliferation. Open Life Sci..

[34] Chang A.L., Miska J., Wainwright D.A., Dey M., Rivetta C.V., Yu D., Kanojia D., Pituch K.C., Qiao J., Pytel P. (2016). CCL2 produced by the glioma microenvironment is essential for the recruitment of regulatory t cells and myeloid-derived suppressor cells. Cancer Res..

[35] Santos B.L., Oliveira M.N., Coelho P.L.C., Pitanga B.P.S., da Silva A.B., Adelita T., Silva V.D.A., Costa M.D.F.D., El-Bachá R.S., Tardy M. (2015). Flavonoids suppress human glioblastoma cell growth by inhibiting cell metabolism, migration, and by regulating extracellular matrix proteins and metalloproteinases expression. Chem.-Biol. Interact..

[36] Victor M.M., David J.M., Cortez M.V.M., Leite J.L., da Silva G.S.B. (2021). A High-Yield Process for Extraction of Hesperidin from Orange (*Citrus sinensis* L. osbeck) Peels Waste, and Its Transformation to Diosmetin, A Valuable and Bioactive Flavonoid. Waste Biomass Valorization.

[37] Victor M.M., David J.M., Sakukuma M.C., França E.L., Nunes A.V. (2018). A simple and efficient process for the extraction of naringin from grapefruit peel waste. Green. Process. Synth..

[38] Lima I.S., Soares É.N., Nonaka C.K.V., Souza B.S.d.F., dos Santos B.L., Costa S.L. (2024). Flavonoid Rutin Presented Anti-Glioblastoma Activity Related to the Modulation of Onco miRNA-125b Expression and STAT3 Signaling and Impact on Microglia Inflammatory Profile. Brain Sci..

[39] Voigt W. (2005). Sulforhodamine B assay and chemosensitivity. Methods Mol. Med..

[40] Papadimitriou M., Hatzidaki E., Papasotiriou I. (2019). Linearity Comparison of Three Colorimetric Cytotoxicity Assays. J. Cancer Ther..

[41] Kramer N., Walzl A., Unger C., Rosner M., Krupitza G., Hengstschläger M., Dolznig H. (2013). In vitro cell migration and invasion assays. Rev. Mutat. Res..

[42] Pohjanvirta R., Niittynen M., Lindén J., Boutros P.C., Moffat I.D., Okey A.B. (2006). Evaluation of various housekeeping genes for their applicability for normalization of mRNA expression in dioxin-treated rats. Chem.-Biol. Interact..

[43] Prokopec S.D., Buchner N.B., Fox N.S., Chong L.C., Mak D.Y.F., Watson J.D., Petronis A., Pohjanvirta R., Boutros P.C. (2013). Validating reference genes within a mouse model system of 2,3,7,8-tetrachlorodibenzo-p-dioxin (TCDD) toxicity. Chem.-Biol. Interact..

[44] Schmittgen T.D., Livak K.J. (2008). Analyzing real-time PCR data by the comparative CT method. Nat. Protoc..

[45] Abotaleb M., Samuel S.M., Varghese E., Varghese S., Kubatka P., Liskova A., Büsselberg D. (2018). Flavonoids in cancer and apoptosis. Cancers.

[46] Chen X.J., Wu M.Y., Li D.H., You J. (2016). Apigenin inhibits glioma cell growth through promoting microRNA-16 and suppression of BCL-2 and nuclear factor-κB/MMP-9. Mol. Med. Rep..

[47] Larigot L., Juricek L., Dairou J., Coumoul X. (2018). AhR signaling pathways and regulatory functions. Biochim. Open.

[48] Rothhammer V., Quintana F.J. (2019). The aryl hydrocarbon receptor: An environmental sensor integrating immune responses in health and disease. Nat. Rev. Immunol..

[49] Jin U.H., Michelhaugh S.K., Polin L.A., Shrestha R., Mittal S., Safe S. (2020). Omeprazole inhibits glioblastoma cell invasion and tumor growth. Cancers.

[50] Bellail A.C., Hunter S.B., Brat D.J., Tan C., van Meir E.G. (2004). Microregional extracellular matrix heterogeneity in brain modulates glioma cell invasion. Int. J. Biochem. Cell Biol..

[51] de Gooijer M.C., Guillén Navarro M., Bernards R., Wurdinger T., van Tellingen O. (2018). An Experimenter’s Guide to Glioblastoma Invasion Pathways. Trends Mol. Med..

[52] Litzenburger U.M., Opitz C.A., Sahm F., Rauschenbach K.J., Trump S., Winter M., Ott M., Ochs K., Lutz C., Liu X. (2014). Constitutive IDO expression in human cancer is sustained by an autocrine signaling loop involving IL-6, STAT3 and the AHR. Oncotarget.

[53] Takacs G.P., Kreiger C.J., Luo D., Tian G., Garcia J.S., Deleyrolle L.P., Mitchell D.A., Harrison J.K. (2023). Glioma-derived CCL2 and CCL7 mediate migration of immune suppressive CCR2+/CX3CR1+ M-MDSCs into the tumor microenvironment in a redundant manner. Front. Immunol..

[54] Qian Y., Dong J., Zhang W., Xue X., Xiong Z., Zeng W., Wang Q., Fan Z., Zuo Z., Huang Z. (2024). Deguelin inhibits the glioblastoma progression through suppressing CCL2/NFκB signaling pathway. Neuropharmacology.

[55] Hwang J.S., Jung E.H., Kwon M.Y., Han I.O. (2016). Glioma-secreted soluble factors stimulate microglial activation: The role of interleukin-1β and tumor necrosis factor-α. J. Neuroimmunol..

